# Bridging brain and blood: a prospective view on neuroimaging-exosome correlations in HIV-associated neurocognitive disorders

**DOI:** 10.3389/fneur.2024.1479272

**Published:** 2025-01-07

**Authors:** Haixia Luo, Junzhuo Chen, Jiaojiao Liu, Wei Wang, Chuanke Hou, Xingyuan Jiang, Juming Ma, Fan Xu, Xire Aili, Zhongkai Zhou, Hongjun Li

**Affiliations:** ^1^Department of Radiology, Beijing Youan Hospital, Capital Medical University, Beijing, China; ^2^Department of Radiology, Qilu Hospital of Shandong University, Jinan, Shandong, China; ^3^Laboratory for Clinical Medicine, Capital Medical University, Beijing, China; ^4^Laboratory for Clinical Medicine, Capital Medical University, Beijing, China

**Keywords:** HIV-associated neurocognitive disorders, neuroimaging, exosomes, biomarkers, early diagnosis

## Abstract

HIV-associated neurocognitive disorder (HAND) is a complex neurological complication resulting from human immunodeficiency virus (HIV) infection, affecting about 50% of individuals with HIV and significantly diminishing their quality of life. HAND includes a variety of cognitive, motor, and behavioral disorders, severely impacting patients’ quality of life and social functioning. Although combination antiretroviral therapy (cART) has greatly improved the prognosis for HIV patients, the incidence of HAND remains high, underscoring the urgent need to better understand its pathological mechanisms and develop early diagnostic methods. This review highlights the latest advancements in neuroimaging and exosome biomarkers in HAND research. Neuroimaging, particularly magnetic resonance imaging (MRI), offers a non-invasive and repeatable method to monitor subtle changes in brain structure and function, potentially detecting early signs of HAND. Meanwhile, exosomes are nano-sized vesicles secreted by cells that serve as key mediators of intercellular communication, playing a crucial role in the neuropathology of HIV and potentially acting as a critical bridge between peripheral blood and central nervous system lesions. Thus, combining plasma exosome biomarkers with indicators derived from neuroimaging scans may enhance the early diagnosis of HAND. This review summarizes evidence supporting the role of exosomes as reliable biomarkers for early detection and management of HAND. Furthermore, we emphasize the correlation between neuroimaging biomarkers and exosome biomarkers and explore their potential combined use. This review discusses the technical challenges and methodological limitations of integrating these two types of biomarkers and proposes future research directions. This multidisciplinary integrative approach not only promises to improve the neurocognitive health management of HIV patients but may also offer valuable insights for research into other neurodegenerative diseases.

## 1 Introduction

Human Immunodeficiency Virus (HIV) infection and its resulting Acquired Immunodeficiency Syndrome (AIDS) remain major global public health challenges. It is estimated that about 39.9 million people worldwide are currently infected with HIV, and approximately 630,000 people died from AIDS-related illnesses globally in 2023 (1). Although combined antiretroviral therapy (cART) has significantly improved patient prognosis, nearly half of the patients who achieve viral suppression through treatment develop HAND (2). HAND remains an urgent clinical problem to be addressed.

HIV has significant neurotropism and can invade the central nervous system (CNS) early in infection. Studies have shown that HIV RNA can be detected in cerebrospinal fluid (CSF) within just 8 days after HIV transmission (3), and magnetic resonance spectroscopy (MRS) examinations can also reveal abnormal brain metabolic information (4). Combination antiretroviral therapy (cART) exhibits multifaceted therapeutic efficacy in HIV infection through distinct mechanistic pathways. In terms of antiviral activity, cART strategically targets key viral enzymatic machinery, including reverse transcriptase, protease, and integrase, effectively disrupting viral replication cycles and suppressing plasma viral load below quantification thresholds (5). Regarding immunological restoration, cART demonstrates significant efficacy in immune system reconstitution, manifested through enhanced CD4+/CD8+ T cell ratios, attenuated immune activation, and diminished pro-inflammatory cytokine production (6, 7).

These therapeutic mechanisms contribute to substantial improvements in clinical outcomes among HIV-infected individuals. In the context of neuropathology, cART administration significantly reduces HIV-associated dementia (HAD) incidence, facilitates synaptic integrity restoration, and enhances neuronal network connectivity (8). Advanced neuroimaging analyses demonstrate that timely cART initiation mitigates cerebral volume reduction and preserves cortical thickness (9), while simultaneously optimizing brain network functionality (10, 11). Remarkably, significant improvements in cognitive function and neural connectivity manifest even within short-term (12-week) therapeutic interventions (12). Longitudinal investigations reveal that sustained therapeutic intervention normalizes cerebral metabolic patterns (13), with particularly pronounced benefits observed during acute HIV infection intervention, facilitating cerebrospinal fluid inflammatory marker normalization (14).

Nevertheless, it warrants emphasis that despite cART’s efficacy in suppressing peripheral viral replication, the central nervous system (CNS) functions as a viral reservoir or “sanctuary site” for HIV, wherein the virus maintains persistent infection within macrophages, astrocytes, and microglial cells (15–17), consequently initiating and perpetuating chronic neuroinflammation. This chronic inflammatory and oxidative stress environment in the brains of people living with HIV (PLWH) leads to neuronal dysfunction and structural changes, ultimately resulting in the occurrence of HAND (2, 6, 8).

The pivotal role of exosomes in HIV pathogenesis has garnered substantial scientific attention in recent years (18–20). These nanoscale vesicles, representing a crucial subpopulation of extracellular vesicles (EVs) with dimensions ranging from 30 to 100 nm, are secreted by diverse cellular populations, including neurons. They orchestrate multiple critical physiological processes, encompassing blood–brain barrier permeability regulation, neurogenesis modulation, intercellular communication facilitation, neuronal stress response mediation, and synaptic plasticity maintenance. Of particular significance, neuron-derived exosomes (NDEs) demonstrate distinctive advantages as potential biomarkers for HIV-associated neurocognitive disorder (HAND): they serve as direct indicators of central nervous system pathological alterations, their constituents specifically reflect neuronal functional status, and their accessibility through peripheral blood enables non-invasive, longitudinal monitoring of central nervous system diseases (21–24).

Integrating neuroimaging with exosome analysis provides a new research direction for the early diagnosis of HAND. This interdisciplinary approach has the potential to bridge the gap between brain pathological changes and peripheral blood biomarkers, leading to breakthrough progress in the diagnosis and treatment of HAND. This review aims to explore the correlation between neuroimaging and exosome biomarkers in HAND research. We will review the application of existing and emerging neuroimaging techniques in detecting HIV-related brain imaging biomarkers and discuss the potential of exosomes, especially NDEs, in HAND diagnosis. By integrating the latest advances in these two fields, we hope to provide new perspectives for the early diagnosis and development of individualized treatment strategies for HAND, ultimately improving the quality of life for PLWH.

## 2 Neuroimaging in HAND

### 2.1 Structural MRI findings in HAND

Since the onset of the HIV epidemic, macrostructural MRI imaging has been widely used to evaluate structural brain changes in PLWH. In conventional T1-weighted MRI imaging, macrostructural neuroimaging can measure changes in gray matter (GM), white matter (WM), and cerebrospinal fluid globally or locally, with gray matter assessment including metrics such as volume, thickness, and surface morphology (4).

With the widespread use of combined antiretroviral therapy (cART), the impact of HIV on central nervous system tissues has gradually diminished (25, 26). Nevertheless, in chronically infected PLWH with suppressed viral loads, voxel-based morphometry (VBM) (27) and surface-based gray matter thickness analysis (SBM) (9, 28) can still detect changes in brain structure. This suggests that even after antiretroviral therapy, there remains a degree of brain structural change in patients with HAND. Notably, it remains unclear whether aging interacts with post-treatment HIV infection, thereby affecting the structural improvements brought about by antiretroviral therapy.

Studies have found that, compared to uninfected controls, virally suppressed PLWH exhibit significant reductions in gray matter volume across multiple brain regions, particularly in the frontal and parietal cortices (29), subcortical structures including the striatum (30, 31), as well as the cingulate gyrus, motor cortex, thalamus, and hippocampal regions (4, 29, 30). These changes are associated with cognitive dysfunction. CD4+ cell counts are significantly correlated with reduced hippocampal and thalamic volumes and increased ventricular volume, while detectable viral loads negatively correlate with hippocampal and amygdalar volumes (32). Cortical and subcortical gray matter volume reductions are more pronounced in patients with a history of non-CNS AIDS-defining illnesses (31).

It has been reported that HIV-positive participants with poorer cognitive abilities have thinner cortices and reduced subcortical volumes, and these changes are associated with increased white matter hyperintensities (33). Recently, Chien et al.’s study further revealed the connection between white matter changes and cognitive function. They found that increases in white matter hyperintensity volume were significantly associated with lower scores in executive function and memory domains (34). HAND patients show increased abnormal white matter, with HAD patients having smaller white matter volumes compared to other control groups (35). ANI patients are associated with specific prefrontal white matter atrophy, while MND shows more widespread atrophy, including lateral ventricle enlargement and white matter atrophy in parietal, frontal, and cingulate regions, with subcortical atrophy correlated with reduced CD4+/CD8+ cell ratios (36).

Longitudinal MRI studies have highlighted the ongoing impact of HIV infection on brain structure. Untreated HIV infection results in significant subcortical atrophy and cortical thinning, while timely cART treatment can effectively prevent these damages (9). However, Liu et al.’s study on SIV-mac239 infected rhesus macaques demonstrated that brain atrophy could still be observed even when cART treatment was initiated immediately after acute infection, although signs of reversal were noted (37). Human brain tissue may continue to suffer damage even when the viral load is undetectable (38). Multiple factors such as viral load, CD4+ cell count, HIV duration, and cognitive decline are linked to brain atrophy (38). These findings underscore the importance of early diagnosis and treatment, as well as the necessity of developing neuroprotective strategies.

Structural MRI has been crucial in revealing neurocognitive disorders and neuroinflammation. Studies have shown that gray matter volume reductions in PLWH are mainly concentrated in the thalamus, prefrontal cortex, parietal, and occipital regions, and these changes are closely related to cognitive dysfunction (34, 39). Reduced thalamic volume and decreased integrity of projection fibers are significantly associated with cognitive decline, possibly due to the thalamus acting as a relay station between subcortical regions and the cerebral cortex (39). However, recent studies have found that in ANI patients, the volumes of the putamen and caudate nucleus are actually increased (40, 41), possibly reflecting the activation of neuroinflammation or compensatory mechanisms. Patients receiving cART treatment show stronger overall information integration capabilities in brain networks, but significantly reduced small-world properties and regional functional segregation (7). Even when the virus is controlled, there are ongoing processes of damage and inflammation in the nervous system. Cerebrospinal fluid analysis reveals significantly elevated levels of neuronal injury markers and inflammatory factors (42). Lower CD4/CD8 ratios and higher proportions of CD16+ inflammatory monocytes correlate with reductions in cortical and gray matter volumes, while elevated sCD14 levels are significantly linked to decreased cerebral blood flow (37, 43).

### 2.2 Diffusion MRI insights into HAND pathology

Diffusion Tensor Imaging (DTI) can detect white matter fiber integrity and microstructural abnormalities by measuring the fractional anisotropy (FA) and mean diffusivity (MD) of fiber bundles, thus providing precise information about neural connections and brain tissue health. Numerous studies have shown that FA and MD are altered in PLWH, especially in subcortical regions such as the basal ganglia and corpus callosum (44, 45). Damage to the corpus callosum can occur within 100 days of HIV infection, followed by damage extending to widespread periventricular white matter areas, including the corona radiata and centrum semiovale (44).

Abnormalities in DTI metrics indicate HIV-related tissue damage and neuroinflammation. Diffusion basis spectrum imaging using tensor models sensitive to cell density found high cell density in PLWH receiving antiretroviral therapy, suggesting persistent inflammation affecting DTI assessment of white matter integrity (46). Despite viral suppression in HIV-infected individuals after combined antiretroviral therapy (cART) treatment, elevated levels of inflammatory biomarkers remain associated with DTI white matter abnormalities and cognitive impairment. These biomarkers include monocyte/macrophage activation markers, chemokines, cytokines, and metalloproteases (47). In identifying HIV-induced brain damage processes through machine learning models, inflammatory markers were identified as discriminative features and quantitatively analyzed using DTI metrics and brain volume measurements (48).

Cross-sectional studies reveal correlations between clinical immune indicators and DTI measurements. Research suggests that HIV-related immunosuppression negatively correlates with white matter integrity (39). In HIV+ samples, higher central nervous system penetration effectiveness of antiretroviral drugs, higher current CD4+ T cell counts, and immune recovery from the lowest CD4+ T cell count are linked to increased FA and decreased MD (49). Additionally, whole-brain analysis using tract-based spatial statistics (TBSS) indicates that a longer duration of CD4 cell counts below 500 cells/ml is associated with lower FA values and higher MD values in commissural, projection, and callosal fibers (50). In SIV-mac239 infected Chinese rhesus macaques, decreased FA and increased MD are detectable in the internal capsule, striatum, brainstem, and corpus callosum 4 weeks post-infection, with these changes worsening over time. However, cART treatment can reverse, alleviate, or enhance these changes, closely related to CD4/CD8 ratios and viral load (51).

Changes in white matter fibers in HAND at the DTI observational level are statistically significant, with longer HIV infection duration and cognitive impairment linked to decreased FA and increased MD (49, 52). HIV-positive individuals with cognitive impairment show significant FA decreases and MD increases in multiple white matter tracts (such as the superior longitudinal fasciculus and mid-corpus callosum), and these white matter damages negatively correlate with enhanced functional connectivity in gray matter regions (53). Even with complete suppression of plasma viral load, HIV-positive patients still exhibit significant changes in brain structural connectivity, especially in occipital and subcortical regions, and these structural connectivity metrics are significantly correlated with complex motor skills (54). Therapies that reduce chronic inflammation and safeguard mitochondrial function might help preserve white matter integrity in older HIV+ individuals (55).

### 2.3 Functional MRI revelations in HAND

Functional Magnetic Resonance Imaging (fMRI) evaluates neuronal activity by measuring Blood Oxygen Level Dependent (BOLD) signals or perfusion changes, offering a crucial complement to structural neuroimaging. BOLD fMRI is primarily categorized into resting-state (RS) fMRI and task fMRI.

Resting-state functional Magnetic Resonance Imaging (rsfMRI), a non-invasive neuroimaging technique, assesses neuronal function by measuring spontaneous low-frequency fluctuations in the brain, serving as a vital tool for studying HAND (56, 57). rsfMRI estimates functional connectivity between regions by analyzing consistent activity across different brain networks during rest, a method extensively used in brain function research (58).

In studies of local brain regions, PLWH exhibit functional changes in areas such as the frontal, occipital, and temporal lobes, and striatal cortex, regions closely linked to learning, memory, and executive functions (59). Research indicates that even at the Asymptomatic Neurocognitive Impairment (ANI) stage, patients display enhanced local activity in visual network areas, the middle frontal gyrus, and cerebellar vermis (60). Furthermore, functional changes in PLWH are mainly detected in the slow-5 frequency band (0.01–0.027 Hz), suggesting this band may be more sensitive to early functional changes in HAND (61).

Recent studies reveal an increased fractional amplitude of low-frequency fluctuation (fALFF) in the occipital cortex and decreased functional connectivity (FC) in the prefrontal cortex of HAND patients (62). They also show enhanced FC between the right superior occipital gyrus and olfactory cortex, emphasizing the role of occipital and prefrontal cortices in HIV-related cognitive dysfunction. However, a large-scale single-center study challenged these views by comparing resting-state functional connectivity (RSFC) in 316 HIV-infected individuals and 209 healthy controls, finding no significant correlations between HIV infection status, viral load, cognitive function status, and RSFC (63), highlighting the importance of large sample size studies.

Despite controversies, other studies have found effects of HIV infection on brain function. rsfMRI studies have revealed connectivity changes in multiple typical brain networks, including the salience network, default mode network (DMN), and executive network (64, 65). Research shows decreased connectivity within the dorsal somatosensory motor network (dSMN) and between dSMN and the medial temporal lobe in PLWH (66). Even in the acute HIV infection (AHI) stage, patients show DMN functional connectivity abnormalities, particularly weakened connections between the left parahippocampal gyrus and left middle frontal lobe, despite intact white matter structure (10).

The protective effects of combined antiretroviral therapy (cART) on brain function have been confirmed. In simian immunodeficiency virus (SIV) models, cART can protect brain function, and DMN network connectivity may serve as a potential biomarker for early detection of viral infection and evaluation of treatment efficacy (67). A prospective longitudinal study further confirms (11) that intensified cART regimens can significantly improve functional connectivity across multiple networks, with this improvement strongly positively correlated with neurocognitive function. Recent research exploring the effects of working memory training on brain functional connectivity in PLWH found that adaptive working memory training can normalize the eigenvector centrality of the ventral default mode network (vDMN) in PLWH, correlating with improved memory performance (68). This provides new insights into HIV cognitive rehabilitation mechanisms and identifies potential therapeutic targets.

To enhance HAND diagnosis and prediction accuracy, researchers have proposed connectome-based predictive models (CPM), combining resting-state FC and white matter structural connectivity (SC) features, and integrating clinical and demographic data to better assess individual cognitive performance in PLWH (69). Overall, the application of rsfMRI in HAND research deepens the understanding of disease neurological mechanisms, providing new directions for early diagnosis, treatment evaluation, and personalized treatment strategy development.

## 3 Exosomes as emerging biomarkers in HAND

### 3.1 The role of exosomes in the pathogenesis of neurodegenerative diseases

The pathological progression of neurodegenerative diseases involves complex intercellular interactions, with a common feature being the misfolding and accumulation of amyloid proteins inside and outside neurons. Exosomes, as crucial mediators of intercellular communication, can transmit signaling molecules between neurons, astrocytes, and microglia (70). Recent studies have highlighted the importance of exosomes in pathological protein transmission, regulation of neuroinflammation, and disease diagnosis (64), offering new perspectives for understanding disease mechanisms and developing novel therapeutic strategies.

Exosomes are nano-sized membrane vesicles released from all cells, including those in the central nervous system (CNS). Similar to retroviruses, they transport bioactive molecules between cells, carrying contents such as RNA, proteins, and lipids. Exosomes are abundantly present in biological fluids like plasma and cerebrospinal fluid (8).

Exosomes are involved in the intercellular transmission of various neurodegenerative disease-related proteins, considered a core mechanism of disease progression (65). Quiroz-Baez et al. noted that EVs from neurodegenerative disease patients contain unique protein markers. These markers, including altered levels of Tau and β-amyloid, offer potential biomarkers for early diagnosis and monitoring of disease progression (71). In Alzheimer’s disease (AD), for example, Aβ-associated exosomes from AD model mice and patients can be absorbed by neurons and target mitochondria, triggering caspase activation and neuronal death. This mechanism was further confirmed by *in vivo* experiments (72). Similarly, in Parkinson’s disease (PD), researchers found α-syn oligomers in microglia-derived exosomes in PD patients’ cerebrospinal fluid. These exosomes can induce α-syn aggregation in recipient neurons, a process further enhanced by inflammatory factors, ultimately leading to degeneration of the nigrostriatal pathway (73). These findings highlight the key role of exosomes in pathological protein transmission.

Exosomes play a crucial role in regulating neuroinflammation and oxidative stress, two processes closely related to various neurodegenerative diseases (73). The significant increase in inflammatory factors (such as IFN-γ, RANTES, GRO) and decrease in growth factors (such as VEGF, FGF-4, EGF) in exosomes reveal enhanced inflammatory responses and reduced neurotrophic support during AD progression (74). M1 microglial-derived exosomes can activate resting microglia and enhance their pro-angiogenic ability. This process involves miR-155-5p-mediated Socs1 inhibition and NFκB pathway activation, triggering inflammatory cascades (75). Studies have shown that activated microglia secrete tau protein-containing exosomes, which can spread to neurons and promote AD progression (76). As AD severity increases, the expression of neuron-derived markers (such as MOG and CD171) in patients’ exosomes significantly rises, reflecting the extent of neuronal damage (74). Meanwhile, the increase in endothelial cell-derived exosomes suggests blood–brain barrier dysfunction. These findings provide a basis for using exosomes as biomarkers of disease progression.

As nano-dimensional vesicular carriers orchestrating intercellular material transport and communication, exosomes play pivotal roles in epigenetic regulation of central nervous system diseases through their capacity to deliver miRNAs and modulate target cell gene expression (77, 78). Exosomal miRNAs influence the progression of neurodegenerative diseases by regulating key signaling pathways involved in neuroinflammation, neuronal survival, and apoptotic processes (79–81).

At the molecular level, BACE1, serving as the primary APP-cleaving enzyme, initiates Aβ formation (82). Exosomal miR-342-5p and the miR-29 family (comprising miR-29a, miR-29b1, and miR-29c) modulate β-amyloid formation through regulation of BACE1 mRNA expression (83–85). Additionally, miR-185-5p regulates APP distribution in exosomes by binding to APP transcript 3’UTR, resulting in upregulated APP expression in recipient cells and subsequent alterations in Aβ production. Notably, miR-185-5p expression is significantly downregulated in serum-derived exosomes from AD patients (86, 87). Regarding neuroinflammation, mesenchymal stem cell-derived exosomal miR-223 attenuates neuronal death by targeting the PTEN-PI3K/Akt signaling pathway (88). Furthermore, exosomal circRNAs function as competing endogenous RNAs, orchestrating gene expression networks through miRNA sponging, thereby playing pivotal roles in the pathological processes of neurodegenerative diseases, including Parkinson’s disease (89). Clinical investigations have revealed that alterations in specific plasma exosomal miRNAs (such as let-7g-5p, miR-126-3p, and miR-142-3p) strongly correlate with disease severity in Alzheimer’s and Parkinson’s diseases, offering potential molecular biomarkers for diagnosis (74, 90, 91). These findings demonstrate that exosomal miRNAs participate in the pathogenesis of neurodegenerative diseases through multiple molecular mechanisms.

As an emerging therapeutic strategy, exosomal miRNAs possess several advantages, including blood–brain barrier penetration, protection from degradation, and simultaneous regulation of multiple disease-related genes. Accumulating evidence suggests the therapeutic potential of various exosomal miRNAs: microglia-derived exosomal miR-124-3p enhances cognitive function by targeting Rela to inhibit β-amyloid deposition (92); bone marrow mesenchymal stem cell-derived exosomal miR-146a promotes hippocampal synapse formation by suppressing astrocyte-mediated inflammation through NF-κB pathway inhibition (93); and MSC-derived exosomal miR-132-3p improves neuronal and synaptic function while reducing cortical and hippocampal Aβ levels via activation of the Ras/Akt/GSK-3β pathway (94). These findings demonstrate that exosomal miRNAs can exert neuroprotective effects through multiple mechanisms, providing compelling experimental evidence for developing novel miRNA-based therapeutic strategies targeting neurodegenerative diseases.

Furthermore, Su et al. demonstrated characteristic lipidomic reconstruction in brain-derived extracellular vesicles (BDEVs) from AD patients, predominantly manifested as diminished levels of polyunsaturated fatty acids and elevated concentrations of specific sphingolipids. Notably, these molecular alterations remained undetectable in total brain tissue analyses, suggesting that exosome-specific lipid signatures could serve as peripheral biomarkers for early-stage Alzheimer’s disease. These findings establish a novel research trajectory for developing exosome-based diagnostic and monitoring strategies for neurological disorders (95).

### 3.2 Molecular pathogenesis of exosome-dependent HAND and HIV-associated comorbidities

As pivotal mediators of intercellular communication, exosomes orchestrate crucial regulatory functions in HIV infection and its associated complications. In the pathogenesis of HAND, these vesicles share cellular signal transduction cascades with HIV, modulating viral entry and egress through the trafficking of viral proteins and regulatory molecules (19, 20). Notably, exosomes derived from HIV-infected cells function both as vectors for viral components and as modulators of disease progression through their capacity to regulate neuroinflammatory responses and immune function (96, 97).

Regarding viral transmission, HIV-infected cells (including microglia, astrocytes, and infected T cells) release exosomes carrying viral proteins (such as Tat, Nef) and viral RNA (98–101). These viral components can be transmitted to uninfected cells via exosomes (102), leading to a “bystander effect.” The presence of viral proteins and RNA in exosomes not only promotes viral spread within the central nervous system (97, 98) but also exacerbates neurotoxicity and neuroinflammation (98, 103).

In terms of neuroinflammation, exosomes can stimulate pro-inflammatory cytokine production and amplify inflammatory responses by activating uninfected glial cells. Research has shown that exosomes released from HIV-infected cells can induce pro-inflammatory factor production in microglia (101) and astrocytes (104). This persistent inflammatory state not only directly impairs neuronal function but also compromises blood–brain barrier integrity, creating a vicious cycle (101, 102). Furthermore, Sampey et al. discovered that TAR RNA-containing exosomes can stimulate the production of pro-inflammatory cytokines such as IL-6 and TNF-β, thereby intensifying inflammatory responses (105).

Exosomes directly affect neuronal function through multiple pathways. Studies have revealed that Tat protein-induced astrocyte-derived exosomes (ADEVs) can carry amyloid-beta (Aβ). These Aβ-carrying exosomes not only lead to alterations in neuronal dendritic structure, synaptic protein imbalance, and synaptic dysfunction, ultimately causing cognitive impairment (106), but also induce astrocyte activation and proliferation. Activated astrocytes produce excessive reactive oxygen species, impair glutamate uptake function, and subsequently disrupt neurovascular unit integrity, ultimately resulting in neuronal death (107). In terms of epigenetic regulation, exosome-carried miR-132 participates in neuronal morphology regulation, with its aberrant expression significantly correlating with neuronal atrophy (108).

Beyond its effects on the central nervous system, HIV-associated exosomes participate in the development of various complications. Regarding the immune system, exosomes modulate T cell function and activity, promoting both immune activation and suppression while participating in antigen presentation, immune tolerance, and immunosuppression processes, significantly affecting immune system function (102). Studies have shown that HIV-infected individuals have a significantly increased risk of cardiovascular disease, with an incidence rate 37% higher than the general population (109, 110). This increased risk may be potentially associated with functional alterations in HIV-related exosomes. Exosomes secreted by cardiac fibroblasts participate in regulating cardiac hypertrophy and fibrosis, while endothelial cell-derived exosomes play crucial roles in angiogenesis and vascular endothelial regeneration (111). Through these pathways, exosomes may exert important regulatory functions in the pathogenesis of HIV-associated cardiovascular diseases.

Regarding metabolism, studies in non-HIV populations have shown that exosomes secreted by adipose tissue macrophages can influence insulin signaling pathways and glucose metabolism through miRNAs such as miR-155 (112). While metabolic disorders are common in HIV-infected patients, the specific mechanisms of exosome involvement in HIV-related metabolic disorders remain unclear and require further investigation.

Exosomes exhibit bidirectional regulatory effects during HIV infection: exosomes from infected cells promote viral replication and transmission, while those from uninfected cells possess immunoprotective functions (113). Research has shown that TAR RNA in exosomes secreted by HIV-1 infected cells promotes cancer cell growth and malignancy by modulating intracellular signaling networks, revealing potential therapeutic targets for HIV-associated cancers (114).

Regarding therapeutic applications, exosomes, as natural nano-scale delivery vehicles, possess excellent biocompatibility and low immunogenicity, capable of carrying therapeutic molecules such as anti-HIV RNA, showing promise as ideal carriers for HIV vaccines (115). Recent research has found that engineered exosomes delivering zinc finger protein-DNA methyltransferase fusion protein (ZPAMt) can specifically bind to HIV-1 promoters and induce DNA methylation, achieving long-term stable HIV-1 suppression across the blood–brain barrier in humanized mouse models (116). In therapeutic strategies for neurological complications, Zhu et al. discovered that inhibiting neutral sphingomyelinase 2 can regulate brain-derived exosome (BDEVs) release and their miRNA composition, improving neuroplasticity, synaptic function, and expression of inflammation-related miRNAs (117). Liu et al. developed Rabies Virus Glycoprotein (RVG)-modified exosomes that specifically target neurons and promote neurogenesis and synaptic plasticity by overexpressing BDNF (brain-derived neurotrophic factor) to activate the BDNF/TrkB/AKT pathway. This targeted delivery system with blood–brain barrier penetration capability provides an innovative therapeutic strategy for HIV-associated neurological complications (118, 119).

### 3.3 Exosomal biomarkers specific to HAND

HAND represents a prevalent complication in HIV-infected individuals. Although cART has effectively reduced the incidence of HAD, it cannot completely prevent the development and progression of HAND (120). The therapeutic efficacy of cART against HIV within the central nervous system remains limited, as most antiretroviral agents demonstrate poor blood–brain barrier penetration due to their high molecular weight and limited lipophilicity. Recent investigations have revealed that specific biomarkers expressed in circulating exosomal proteins may correlate with viral reactivation and neuropathology, potentially elucidating the etiology of neurological manifestations (136). These findings highlight the potential of NDEs as novel biological markers for disease monitoring and progression assessment.

Multiple studies have confirmed exosomal proteins as potential biomarkers for HAND. Sun et al. found significantly elevated levels of HMGB1, NF-L, and Aβ proteins in NDEs of HIV-infected individuals with cognitive impairment (121, 122). These findings suggest that NDEs contents can reflect neuronal health status, providing new insights for early HAND diagnosis. Additionally, Dagur et al. observed a significant increase in NDEs in the brain and serum of HIV-1 transgenic rats, particularly L1CAM+ NDEs in circulation, suggesting a potential mechanism for peripheral HAND biomarkers (123).

Notably, Guha et al. found that increased abundance of EVs in the cerebrospinal fluid of cognitively impaired HIV-positive individuals positively correlated with the neuronal injury marker neurofilament light chain(NFL). Elevated levels of neuroinflammatory proteins HMGB1 and NLRP3 in NDEs were associated with neuropsychological scores (124, 125). Pulliam et al. used machine learning algorithms to reveal that elevated levels of HMGB1 and NFL proteins in NDEs are important predictors of cognitive deficits (126).

Furthermore, de Menezes et al. found that exosomes expressing neuroinflammatory markers such as CD14, CD16, CD192, CD195, and GFAP are significantly associated with cognitive impairment severity (127). Johnston et al. demonstrated that specific EV subgroups can effectively predict neurocognitive disorders in older HIV-infected individuals, with increased levels of CCR5+ EVs positively correlating with cognitive impairment (128). This study was the first to establish an association between exosomal surface proteins and HAND severity, providing new ideas for clinical stratified diagnosis.

HIV viral proteins, particularly Nef and Tat, demonstrate crucial mechanistic involvement in the pathogenesis of HIV-associated neurocognitive disorder (HAND). Experimental investigations have elucidated that Nef protein orchestrates HAND development through multiple exosome-mediated molecular cascades. Mahfuz et al. demonstrated that Nef-containing exosomes facilitate enhanced expression and secretion of β-amyloid protein (Aβ) and its peptide derivatives in target cells, consequently amplifying HAND severity (129). Complementary investigations by Sami Saribas et al. revealed that neuronal internalization of these Nef-bearing exosomes precipitates oxidative stress and attenuates neuronal action potential generation (98). Of particular significance, clinical investigations by Caobi et al. established a positive correlation between cerebrospinal fluid Nef-containing exosome concentrations and both cognitive dysfunction severity and CD4 T cell quantification. Subsequent proteomic analyses of circulating exosomes revealed distinctive signaling pathway engagement and biological functions in asymptomatic neurocognitive impairment (ANI) and mild neurocognitive disorder (MND) (130), establishing novel biomarker candidates for HAND stratification.

Furthermore, Tat, another critical HIV protein, modulates cognitive function through exosome-dependent mechanisms. Investigations by Chandra et al. demonstrated that Tat-containing exosomes induce mitochondrial dysfunction and oxidative stress in brain endothelial cells, consequently compromising cognitive function (99). These findings were subsequently validated by Henderson et al., who identified biologically active Tat protein within cerebrospinal fluid exosomes, further substantiating its mechanistic role in neurotoxicity (106).

Regarding RNA biomarkers, DeMarino et al. unveiled a critical finding: under antiretroviral therapy, EVs in CSF and serum of HIV-infected individuals persistently harbor viral RNA. Notably, CSF HIV RNA levels demonstrated a significant correlation with HAND, providing pivotal insights into virus-induced neurological damage mechanisms (131). Integrative multi-omics analysis revealed approximately 1,000 differentially expressed genes between HIV-1 infected patients and cognitively normal individuals, predominantly concentrated in miRNA and long non-coding RNA (lncRNA) domains. Enrichment analyses further elucidated that these molecular signatures participate in critical regulatory networks, including neuroinflammation, autophagy, neurogenesis, and mitochondrial dynamics (132). Particularly noteworthy, the lncRNA RP11-677M14.2 (Nrgn-AS) exhibited elevated expression in HIV-1 infected brain tissues. By modulating neurogenin (Nrgn) expression, this lncRNA is proposed as a key determinant in deciphering synaptic injury mechanisms associated with HAND (133). In primate model investigations, researchers observed significant alterations in RNA profiles within extracellular vesicles during SIV infection, with RNA species including mRNA, miRNA, and circRNA predominantly clustered around inflammatory regulation and immune response-related gene networks (8). Furthermore, circRNA, lncRNA, and miRNA carried by extracellular vesicles participate in competitive RNA regulatory networks underlying neurodegenerative disease pathogenesis, offering crucial preliminary insights for early diagnostic and therapeutic strategies (134).

Regarding RNA markers, DeMarino et al. found that EVs in cerebrospinal fluid and serum of ART-treated HIV-infected individuals still contained HIV RNA despite controlled viral replication, with CSF HIV RNA levels associated with neurocognitive dysfunction (131). Huang and colleagues demonstrated that exosomes may participate in SIV infection-related neuropathological processes through the transportation of specific RNA molecules. During SIV infection, expression profiles of various RNA species in BDEVs, including mRNA, miRNA, and circRNA, underwent significant alterations, predominantly involving genes implicated in inflammatory modulation and immunological response pathways (8).

In conclusion, exosomes disrupt neurological function in HAND by spreading inflammatory factors, inducing neurotoxicity, affecting synaptic protein expression, and causing neuronal shrinkage. Exosomes and their contents have great potential as HAND biomarkers. Future research should further explore exosomes’ role in HAND pathogenesis and develop exosome-based diagnostic and therapeutic strategies to improve neurocognitive function in HIV-infected individuals.

## 4 Bridging neuroimaging and exosomal biomarkers in HAND

### 4.1 Convergence of exosome biology and brain imaging in neurodegeneration

Exosomes and exosome-enriched EVs play a crucial role in the pathogenesis of HAND, particularly in neuroinflammation and neurotoxicity. Recent research has revealed their potential as both biomarkers and pathogenic factors. Agliardi et al. found that peripheral blood exosomes derived from the central nervous system (CNS) carry disease-specific molecular markers, potentially reflecting the inflammatory and toxic state of the CNS (135).

In a SIV-infected rhesus macaque model, Chandra et al. observed significantly elevated levels of proteins associated with neuroinflammation and neuropathology in exosomes from SIV-infected monkeys, indicating the role of exosomes in spreading and exacerbating neuroinflammation (136). Huang et al. further revealed the importance of exosomes in regulating neuroinflammatory networks through brain-derived exosome RNA analysis (8). András et al. found that HIV infection increased the number of EVs released by brain microvascular endothelial cells and altered their amyloid β (Aβ) levels, potentially exacerbating neurotoxicity (137). András and Toborek further identified that EV-associated Serpine-1 could reduce synaptic protein expression in neural progenitor cells, directly linking to neurotoxicity and synaptic damage (138). Research by Rahimian, He, and colleagues more directly demonstrated the role of exosomes in neurotoxicity. They found that Tat-expressing astrocytes in HIV-infected individuals release exosomes containing miR-132, which, when absorbed by neurons, leads to neuronal shrinkage and increased neurotoxicity (108). This reveals how exosomes mediate harmful interactions between glial cells and neurons, exacerbating neurotoxicity.

These findings deepen our understanding of exosome-mediated neuroinflammation and neurotoxicity mechanisms in HAND. Multiple studies mentioned above indicate that the contents of neurogenic exosomes in PLWH plasma reveal neuroinflammation in the central nervous system of HIV patients. There is ample evidence that persistent chronic neuroinflammation, neurotoxicity, and oxidative stress lead to HAND, with the neuroinflammatory process likely being central to HAND (123, 139, 140). Subtle changes in brain structure and function caused by HIV infection-induced tissue damage and neuroinflammation can be detected through neuroimaging, which also alters the expression and release of neurogenic exosome contents (5). Therefore, the biological basis for the correlation between neuroimaging and exosome markers lies in the common pathways of neuroinflammation and neurodegenerative changes.

Thus, integrating MRI and exosome analysis in HAND research provides a promising frontier for early diagnosis and mechanistic understanding. In recent years, the integrated analysis of exosomes and magnetic resonance imaging (MRI) has made significant progress in neurological disease research, offering new insights for exploring the pathological mechanisms and diagnostic strategies of HAND. Although HAND research in this field is not yet in-depth, related findings in other neurological diseases provide valuable inspiration.

In other neurodegenerative diseases, studies have observed correlations between brain atrophy detected by MRI and exosomal biomarkers (141, 142). In AD, levels of insulin signaling pathway proteins in neurogenic exosomes were significantly correlated with the degree of atrophy in the temporal, parietal, and prefrontal cortices (99). Researchers believe these findings indicate that exosomal biomarkers reflect ongoing pathological changes in the brain and correspond to structural changes detected by MRI. This finding inspires us to consider whether there might also be a correlation between brain changes detected by MRI and exosomal biomarkers in HAND. Such potential associations could provide new avenues for early diagnosis and progression monitoring of HAND.

The clinical implementation of integrated diagnostic approaches combining neuroimaging techniques with exosomal biomarkers for neurodegenerative disease assessment remains in its developmental phase. A pioneering prospective investigation (ChiCTR2000029055) has been designed to recruit 210 subjects (comprising equal cohorts of 70 participants each in Alzheimer’s disease, amnestic mild cognitive impairment, and control groups) to elucidate the associations between plasma exosomal neurogranin profiles, cognitive function parameters, and neuroanatomical characteristics (143). This longitudinal three-year observational study will provide pivotal clinical evidence for validating the efficacy of this integrated diagnostic paradigm in early-stage neurodegenerative disease detection. Nevertheless, the current paucity of analogous investigations indicates that this research domain remains in its exploratory phase, highlighting the imperative need for additional clinical trials to establish diagnostic accuracy and reliability.

Emerging evidence suggests that exosomal biomarkers serve as molecular indicators of neurodegenerative structural modifications in the brain, establishing a novel paradigm for investigating neurodegenerative disorders. Kumar and colleagues demonstrated significant associations between chronic opioid-induced regional gray matter atrophy and elevated concentrations of neurofilament light chain (NFL) and α-synuclein in circulating exosomes (144). Considering the elevated prevalence of opioid misuse among HIV-infected populations and its documented potential to amplify HIV-associated neurological deterioration (145, 146), this innovative methodological integration of exosomal profiling with neuroimaging analyses may provide unprecedented insights into HAND pathogenesis and disease trajectory.

Recently, Zhang et al. found in a type 2 diabetes rat model that treatment with brain endothelial cell-derived exosomes could significantly improve neurovascular function (147). Through MRI analysis, they observed significant increases in cerebral blood flow (CBF) in the corpus callosum and hippocampus, and decreased blood–brain barrier permeability in the cerebral cortex after exosome treatment. This finding has important implications for HAND research, as HIV infection often leads to blood–brain barrier dysfunction and changes in the neurovascular unit. We can speculate that in HAND patients, a decrease in brain endothelial-derived exosomes might lead to decreased neurovascular function in HIV-susceptible brain regions such as the prefrontal cortex and basal ganglia through similar mechanisms.

Zhan et al.’s research found that exosomes derived from adipose-derived stem cells (ADSCs) can provide neuroprotection by regulating hippocampal cell pyroptosis (148). Using fMRI technology, they observed that exosome treatment significantly improved functional connectivity between the hippocampus and other brain regions (such as the striatum and thalamus). These findings are significant for HAND research, as hippocampal dysfunction is a common cause of cognitive impairment in HAND patients. We can envision that in HAND patients, a decrease in ADSC-derived exosomes may correlate with memory and learning deficits caused by hippocampal dysfunction.

Integrating exosome biology with neuroimaging opens new prospects for HAND research. This innovative approach, combining brain structural and functional imaging with exosome molecular levels, has the potential to revolutionize our understanding of HAND pathogenesis (104, 139). It could enable early diagnosis and intervention for HIV patients potentially progressing to HAND and provide more precise, individualized strategies for diagnosis, treatment, and prognosis evaluation.

### 4.2 Challenges and prospects in HAND biomarker integration

The integration of neuroimaging and exosome biomarkers has opened a promising new field for research on HAND, providing unprecedented insights into disease mechanisms and the development of early diagnostic tools. However, this emerging field faces significant challenges that must be addressed to fully realize its potential.

Of primary consideration, Contemporary methodological constraints in exosome isolation and characterization frequently generate heterogeneous extracellular vesicle populations, particularly manifesting in miRNA compositional diversity. This heterogeneity predominantly originates from dual mechanisms: the selective orchestration of miRNA incorporation into exosomes and the diverse cellular derivation of exosomal populations. The sorting paradigm encompasses both selective mechanisms predicated on specific protein-RNA interactions and non-selective pathways correlating with cytoplasmic miRNA abundance. Furthermore, exosomal cargo composition demonstrates significant plasticity across cellular phenotypes, physiological states, and environmental conditions (149, 150). This inherent heterogeneity substantially compromises the specificity and reliability of exosomal miRNAs as potential diagnostic biomarkers (79).

Additionally, methodological variations in neuroimaging techniques and their interpretative frameworks introduce further complexity to interdisciplinary integration efforts (151). The absence of standardized protocols presents substantial impediments to cross-study comparative analyses (152). Concurrently, the heterogeneous clinical manifestations of HAND and multiple confounding variables further complicate data interpretation. Considering HIV’s multifaceted impact on the central nervous system, a sophisticated integrative approach is imperative for comprehensive biomarker analysis in understanding HAND pathogenesis and progression.

To address these methodological challenges, future investigative efforts should prioritize the development of refined exosome isolation strategies, particularly emphasizing neuron-derived exosome isolation through specific molecular markers such as L1CAM (153). Advanced computational approaches, including machine learning algorithms, can enhance detection precision, while integrated multi-omics analyses may provide more comprehensive mechanistic insights. The standardization of neuroimaging protocols and establishment of unified analytical frameworks are fundamental prerequisites for facilitating meaningful cross-study comparative analyses.

Despite these challenges, recent studies have revealed important links between exosome markers and neuroimaging changes in HAND. In terms of brain structure, research has found gender-specific differences in protein expression in plasma NDEs of HIV-infected individuals, potentially explaining gender-specific brain structural changes observed in T1-weighted MRI (122, 154). András et al.’s study suggests that HIV infection affects brain endothelial exosome release and amyloid-β (Aβ) levels, providing new perspectives on blood–brain barrier dysfunction and neuroinflammation (107, 137).

In white matter structure research, Li et al. found that M2 microglia-derived exosomes promote white matter repair through miR-23a-5p, revealing potential links between white matter integrity and exosome-carried myelin-related proteins or miRNAs (155). This provides new mechanistic insights into understanding white matter microstructural changes in HAND.

In brain functional connectivity research, HAND often manifests as alterations in multiple large-scale networks (140). Ku et al. discovered that HIV-1 Tat-induced astrocyte EV miR-7 release may lead to synaptic changes, explaining network functional abnormalities observed in resting-state fMRI (156). The role of Aβ-carrying exosomes in synaptic degeneration and HAND-related behavioral changes further emphasizes the multiple roles of exosomes in HIV-related neuronal damage (100).

Applying machine learning and artificial intelligence to integrated data analysis represents an exciting frontier with the potential to reveal complex patterns difficult to detect using traditional methods (157). Long-term follow-up studies are crucial for understanding disease progression and identifying early markers (21).

Integrating these biomarkers has enormous potential for HAND research and clinical practice, potentially enabling more accurate early diagnosis and personalized treatment (5). However, establishing standardized protocols is crucial for comparing results across studies and building a coherent understanding of HAND pathogenesis (158).

Despite the challenges, this field holds great promise. As research progresses, this interdisciplinary approach may lead to major breakthroughs, not only improving clinical management of HIV-infected individuals but also providing valuable insights for research into other neurodegenerative diseases. Continued exploration will deepen our understanding of how HIV affects the brain and develop more precise diagnostic and treatment strategies for HAND patients.
